# Activation of glucagon-like peptide-1 receptor in microglia attenuates neuroinflammation-induced glial scarring via rescuing Arf and Rho GAP adapter protein 3 expressions after nerve injury

**DOI:** 10.7150/ijbs.68974

**Published:** 2022-01-16

**Authors:** Zhanyang Qian, Hongtao Chen, Mingjie Xia, Jie Chang, Xinyu Li, Suhui Ye, Shunjie Wu, Shuai Jiang, Junping Bao, Binyu Wang, Renyi Kong, Sheng Zhang, Shengnai Zheng, Xiaojian Cao, Xin Hong

**Affiliations:** 1 Spine center, Zhongda Hospital of Southeast University, Nanjing, China; 2 School of Medicine, Southeast University, Nanjing, China; 3 Department of Orthopedics, The Affiliated Drum Tower Hospital of Nanjing University Medical School, Nanjing, China; 4 Department of Orthopedics, Nanjing First Hospital, Nanjing Medical University, Nanjing, China; 5 Department of Orthopedics, First Affiliated Hospital of Nanjing Medical University, Nanjing, China; 6 School of Health Economics Management, Nanjing University of Chinese Medicine, Nanjing, China

**Keywords:** Spinal cord injury, exendin-4, glucagon-like peptide-1 receptor, inflammation, Arf and Rho GAP adapter protein 3

## Abstract

**Rationale:** The neuroinflammation is necessary for glial group initiation and clearance of damaged cell debris after nerve injury. However, the proinflammatory polarization of excessive microglia amplifies secondary injury via enhancing cross-talk with astrocytes and exacerbating neurological destruction after spinal cord injury (SCI). The glucagon-like peptide-1 receptor (GLP-1R) agonist has been previously shown to have a neuroprotective effect in neurodegeneration, whereas its potency in microglial inflammation after SCI is still unknown.

**Methods:** The effect and mechanism of GLP-1R activation by exendin-4 (Ex-4) were investigated in *in vitro* cultured glial groups and *in vivo* in SCI mice. Alterations in the gene expression after GLP-1R activation in inflammatory microglia were measured using mRNA sequencing. The microglial polarization, neuroinflammatory level, and astrocyte reaction were detected by using western blotting, flow cytometry, and immunofluorescence. The recoveries of neurological histology and function were also observed using imaging and ethological examinations.

**Results:** GLP-1R activation attenuated microglia-induced neuroinflammation by reversing M1 subtypes to M2 subtypes *in vitro* and *in vivo*. In addition, activation of GLP-1R in microglia blocked production of reactive astrocytes. We also found less neuroinflammation, reactive astrocytes, corrected myelin integrity, ameliorated histology, and improved locomotor function in SCI mice treated with Ex-4. Mechanistically, we found that Ex-4 rescued the RNA expression of Arf and Rho GAP adapter protein 3 (ARAP3). Knockdown of ARAP3 in microglia reversed activation of RhoA and the pharmacological effect of Ex-4 on anti-inflammation *in vitro*.

**Conclusion:** Ex-4 exhibited a previously unidentified role in reducing reactive astrocyte activation by mediation of the PI3K/ARAP3/RhoA signaling pathway, by neuroinflammation targeting microglia, and exerted a neuroprotective effect post-SCI, implying that activation of GLP-1R in microglia was a therapeutical option for treatment of neurological injury.

## Introduction

Mechanical injury in spinal cord injury (SCI) causes the appearance of activated microglia *in situ*, followed by the initiation of neuroinflammation and neighboring glial migration 1,2. In the acute phase of secondary injury, these cells produce various proinflammatory cytokines to intensify neuroinflammatory responses 3,4. The uncontrollable neuroinflammation initiated by activated microglia elicits a prominent secondary injury cascade including demyelination, the loss of neurons, and glial accumulation around the originally injured site associated with irreversible neurological functional disorders 5. Microglial polarization has been shown to be a promising treatment in the inflammatory regulation of neurological diseases 6-8. Traditionally, activated microglia contain inflammation-induced “M1” and immunoregulatory “M2” subtypes 9,10. The effective transformation between M1 and M2 microglia plays a positive role in neuroinflammation-related pathological events 11-13. The activation of reactive astrocytes depends on extracellular secreted factors of type M1 microglia 14,15. However, it is an open question whether modulating microglial phenotypes influences the cross-talking with astrocytes and neuronal survival.

Glucagon-like peptide-1 (GLP-1) is an endogenous enteric peptide that facilitates insulin secretion as a function of enteric glucose levels 16. GLP-1R agonist exerts a protective effect on Alzheimer's disease and Parkinson's disease 17-19 using its ability to traverse the blood-brain barrier 20,21. Given the early findings and pharmacological superiority of GLP-1R agonist in neurological pathophysiology, we hypothesized that pharmacological activation of GLP-1R in the specific cells of neural tissue mitigated secondary injury post SCI.

In the present study, we characterized GLP-1R expression in microglia, neurons, and astrocytes, and further established the potential therapeutic value of exendin-4 (Ex-4) in the SCI model during the recovery phase. For the first time, we found a novel pathway for pharmacological regulation of Ex-4 to microglial inflammatory differentiation via GTPase and Ras protein signaling. Microglia-astrocyte co-cultures have identified the mechanism of Ex-4-reduced astrocyte activation, which involved inhibiting the cross-talk of induced microglia. Furthermore, we also showed that microglia exhibited active neuroinflammation and increased glial activation in injured cords, whereas a lack of such pathology following Ex-4 treatment showed a positive modulation on neural structural damage and motor function recovery during post SCI, suggesting a possible therapeutic intervention for SCI.

## Methods

### Extraction and Treatment of Primary Glial Cells

Fresh brains of C57BL/6J neonatal mice (1-day-old) were placed into pre-cooled Dulbecco's Modified Eagle Medium (DMEM; KeyGEN, Nanjing, China). After cleaning pia mater and vessels, we used 0.25% trypsin-EDTA (NCM Biotech, Suzhou, China) to dissolve the brain cortex at 37 °C for 5 min. The cells were seeded in DMEM containing 10% fetal bovine serum (FBS; Gibco, Grand Island, NY, USA) and cultured for 14 days at 37 °C. The cells were then shaken at 37 °C using a table concentrator and the upper microglia were collected after 6 h. The astrocytes then were purified for subsequent studies as described previously 22. The primary microglia and astrocytes were identified using IF and flow cytometry techniques (Figure S1A-C). A total of 1 μg/mL of lipopolysaccharide (LPS; Sigma-Aldrich, St. Louis, MO, USA) was used to activate microglia for 12 h. Ex-4; (MedChemExpress, Shanghai, China) was used for treatment for 24 h.

### RNA-seq Analysis

Total RNA from microglia was extracted by TRIzol reagent (YiFeiXue Tech, Nanjing, China) in accordance with the manufacturer's instruction. The raw data were filtrated using fastp software. After ribosomal RNAs were eliminated, the prepared RNA-seq libraries were sequenced using an Illumina Novaseq 6000 sequencer (Biomarker Technologies, Beijing, China). The expression of genes was determined by Tophat and Cufflinks according to our previous report 23. Gene fusion events in the transcriptome were shown as a fusion-map. GO database annotations with DAVID were used to perform enrichment analysis. The difference of gene pathways was assessed by KEGG (Kyoto Encyclopedia of Genes and Genomes). A *P* value < 0.05 and fold change ≥1 were deemed as screening criteria for the examination of gene expression.

### Transfection of ARAP3 Interference (ARAP3i)

After the microglia confluence reached more than 30%, microglia were incubated with shRNA-ARAP3 (10^7^ TU/mL, Genechem, Shanghai, China) loaded in lentivirus (LV) supplemented 1× HitransG A reagent (Genechem) in complete medium for 12 h. The complete medium then was cultured with cells for 60 h.

### Co-culture of Microglia and Astrocytes

As shown in Fig. 4A, the primary astrocytes were seeded into 24-well plates (Corning, Corning, NY, USA), and microglia undergoing specific treatment in each group were inoculated in the upper inserts (Corning). After 48 h incubation, the medium and astrocytes were collected for subsequent experiments.

### The Spinal Cord Injury (SCI) Model

C57BL/6J adult mice (males, average weight of 20 g, 8 weeks of age) were used for the SCI model. Briefly, mice were anesthetized intraperitoneally using ketamine (80 mg/kg)/xylazine (4 mg/kg) and a moderate contusion injury (5 g × 5 cm) was performed at the 10^th^ thoracic cord using a spinal cord impactor (RWD, Shenzhen, China). Afterwards, the overlying fascia and skin were sutured, and the mice were placed on a temperature-controlled heating blanket until they were conscious. Then intraperitoneal administration of Ex-4 (20 μg/kg) according to a previous study 24, was carried out once a day, for 7 days post SCI.

### Behavioral Assessment

Locomotor functional evaluation used the Basso Mouse Scale (BMS) test 25. Briefly, all animals freely moving in an open field were observed by two blinded researchers and evaluated at 1, 3, 7, 14, 21, and 28 dpi. The swimming test and footprint assay were conducted as previously described 26,27.

### Western Blotting

Total cellular and tissue proteins were extracted using a Total Protein Extraction Kit (KeyGEN) according to the manufacturer's instructions. Equivalent proteins were resolved using 10% SDS-PAGE (EpiZyme, Shanghai, China). The relevant primary and secondary antibodies used in western blotting analyses were the following: anti-iNOS (1:250, ab15323; Abcam, Cambridge, MA, USA), anti-COX-2 (1:1000, ab15191; Abcam), anti-GLP-1R (1:1,000, ab218532; Abcam), anti-Mannose Receptor/CD206 (1:1,000, ab64693; Abcam), anti-ARAP3 (1:1,000, 39906; Singalway Antibody, College Park, MD, USA), anti-Phospho-PI3K (1:1.000, 340790; ZEN BIO, Chengdu, China), anti-PI3K (1:1.000, R22768; ZEN BIO), anti-glyceraldehye 3-phosphate dehydrogenase (GAPDH; 1:10,000; HRP-60004; Proteintech, Rosemount, IL, USA), anti-β-actin (1:10,000, HRP-60008; Proteintech), anti-β-tubulin (1:10,000, HRP-66240; Proteintech), and horseradish peroxidase (HRP) goat-anti-rabbit/mouse IgG (1:10,000; YiFeiXue Biotechnology, Nanjing, China).The proteins of interest were measured using the enhanced chemiluminescence method and quantitative analysis used ImageJ software (National Institutes of Health, Bethesda, MD, USA).

### The RhoA Activation Assay

Cells and tissue were harvested in lysis buffer following the manufacturer's protocol (BK-036, Cytoskeleton, Denver, USA), and centrifuged at 4°C. The supernatant then was incubated with 50 µg rhotekin-RBD beads to pull-down GTP-RhoA at 4°C for 1 h. After washing with wash buffer, the bead samples were resuspended in Laemmli sample buffer and boiled. The proteins on the beads were analyzed using western blots after SDS-PAGE. A total of 1 mM GDP or 0.1% GTPγS, as a positive control or negative control, respectively, were preloaded onto small GTPases by incubating with cytosol for 15 min at room temperature.

### ELISAs

Cell culture medium and fresh cord tissue (4 mm around foci) homogenates were centrifuged at 4 °C. The levels of TNF-α, IL-1β, IL-6, and IL-1α were determined using ELISA Kits (YiFeiXue Biotech; BioLegend, San Diego, CA, USA) following the manufacturer's instructions. The absorbance was then determined at 450 nm using a microplate reader (BioTek, Winooski, VT, USA).

### The Flow Cytometry Assay

The primary microglia and astrocytes were incubated with F4_80-PE (1:500, 565410; BD Biosciences, Franklin Lakes, NJ, USA) or glial fibrillary acidic protein (GFAP)-PE (1:500, 561483; BD Biosciences) for 30 min at 4 °C. For polarization analysis, treated microglia were collected and added to F4_80-PE (565410, BD Biosciences), iNOS-FITC (1:500, 610330; BD Biosciences), and CD206-APC (1:500, 17-2061-82; Thermo Fisher Scientific, Waltham, MA, USA) for incubation. Microglia were loaded in a flow cytometer (FACSVerse 8, BD) and the polarization was visualized. The obtained data were analyzed using FlowJo software (Version 7.6.1; FlowJo, Treestar, OR, USA).

### The Transwell Assay

Primary astrocytes (1 × 10^5^) were resuspended in 200 μL FBS-free DMEM and seeded into the upper inserts (Corning). Media with microglia (MG) in each group was collected after 24 h of culture, then added in a volume ratio of 1:1 with complete DMEM in each of the lower layers, which underwent 24 h of culture. The astrocytes in the upper inserts were fixed with 4% paraformaldehyde (PFA; Servicebio, Wuhan, China) followed by staining with Crystal Violet reagent (KeyGen). We observed cells under the permeable membrane using a microscope (DM2000, Leica, Wetzlar, Germany).

### Immunofluorescence (IF) and Immunohistochemistry (IHC)

The collection of spinal cord tissues and the preparation of paraffin sections were conducted as previously described 28. For cell IF, microglia and astrocytes were first treated with 4% PFA for 15 min. The sections and cells were blocked with Immunol Staining Blocking Buffer (Beyotime) for 1 h and incubated with the primary antibodies overnight at 4 °C, then were incubated with Alexa Fluor secondary antibodies (Jackson ImmunoResearch, PA, USA) for 1 h. This was followed by incubation with a diamidinyl phenyl indole (DAPI) coloration for 10 min (Servicebio). For IHC, the sections were probed with the primary antibodies overnight at 4 °C, incubated with the HRP secondary antibody for 1 h, then reacted with diaminobenzidine, and counterstained with hematoxylin for 5 s. Immunohistochemistry (IHC) and IF staining was performed using the following primary antibodies: anti-NeuN (1:100, MAB377; Millipore, Burlington, MA, USA); anti-GFAP (1:600, 3670; Cell Signaling Technology); anti-IBA-1 (1:500, ab178847; Abcam), anti-GLP-1R (1:500), anti-TNF-α (1:100, GB13452, Servicebio), anti-IL-1β (1:300, GB11113, Servicebio); anti-Nestin (1:200; ab11306; Abcam), anti-iNOS (1:100) and anti-CD206 (1:1,000); anti-ARAP3 (1:200) anti-NF-200 (1:200, ab82259; Abcam); anti-IL-1α (1:200, 16765-1-AP; Proteintech); and anti-MBP (1:600, ab7349; Abcam). Images were captured using a microscope (DM2000; Leica).

### Nissl, Luxol Fast Blue (LFB), and hematoxylin-eosin (HE) staining

To measure the number of neurons and the integrity of the myelin sheath, Nissl and LFB staining of spinal cord were performed using the Nissl Staining Reagent (Servicebio) and LFB Staining Reagent (Servicebio). Tissue HE staining was conducted using an HE Staining Reagent (Servicebio) according to the manufacturer's directions. After mounting, the sections were observed using a microscope.

### Electron Microscopy Imaging (EMI)

Fresh spinal cords at 28 dpi were removed from mice and placed in glutaraldehyde for 24 h. Cords were prepared for electron microscopy and visualized using a transmission electron microscope (Tecnai 12; Philips, Best, Netherlands). The G-ratio of axons (axon diameter/filament diameter) was evaluated according to a previous description 29.

### Micro Magnetic Resonance Imaging (mi-MRI)

Mi-MRI measurement was used to visualize injured neural tissue repair at 28 d post-injury (dpi). After inhalation anesthesia with 2% halothane in 0.4 L/min oxygen and 0.6 L/min nitrogen, the mice were fixed in a small animal mi-MRI system (Bruker BioSpec 7 T/20 USR; Bruker AXS, Karlsruhe, Germany). Parameters were adjusted as previously described 30. T2-weighted images were collected using ParaVision (Version 6.01; BrukerBioSpec; Bruker AXS).

### Statistical Analysis

Data are shown as the mean ± standard deviation (SD) values, and were analyzed using Prism software, version 8.0 (GraphPad, San Diego, CA, USA). Comparisons between two groups were performed using unpaired two-tailed Student's *t*-tests, and among more than two groups using one-way or two-way analysis of variance followed by Tukey's post hoc test. A value of *p* < 0.05 was assumed to be statistically significant.

## Results

### The Agonistic Action of Ex-4 on GLP-1R Reverses Microglia Polarization and Inflammatory Effectors

**Figure 1:**
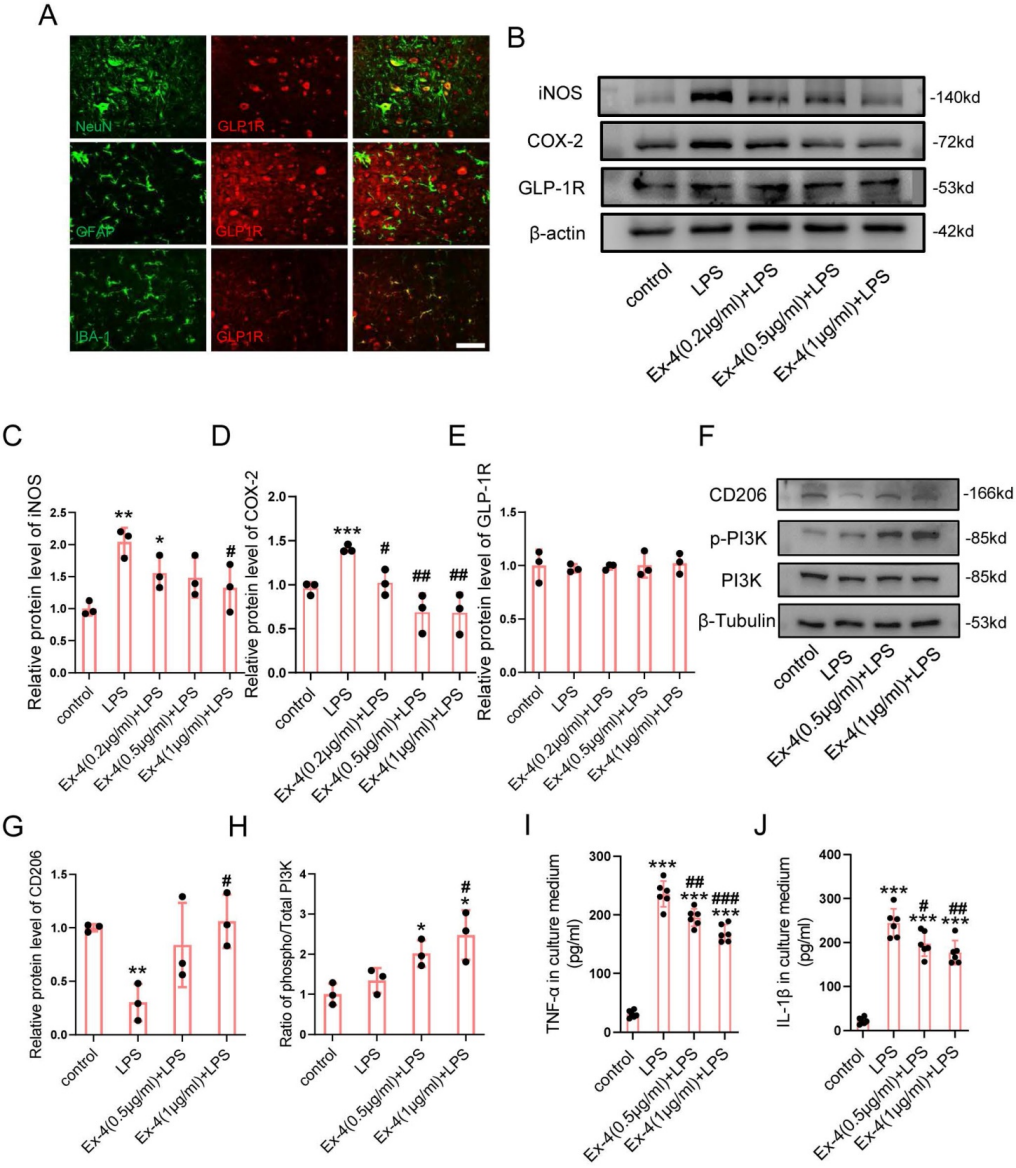
**Activation of GLP-1R reverses microglia polarization and inflammatory effectors. (A)** Representative immunofluorescence labeling images for NeuN, GFAP, and IBA-1 (green) co-staining with GLP-1R (red) obtained from longitudinal sections of normal cords. The blue staining indicates the DAPI-stained nuclei. Scale bar = 100 μm. **(B)** Western blotting including iNOS, COX-2, and GLP-1R in LPS-stimulated primary microglia with treatment of Ex-4 ranging from 0.2 μg/mL to 1μg /mL for 12h; n=3. β-actin was used as the control.** (C)** Bar graph showing the densitometry analysis of iNOS expression.** (D)** Densitometric analysis of COX-2 expression.** (E)** Densitometric analysis of GLP-1R expression. **(F)** Western blotting performed for the proteins including CD206, p-PI3K, and PI3K in LPS-activated microglia with treatment of Ex-4 at doses of 0.5 and 1μg /mL for 12h; n=3. β-Tubulin was used as the control. **(G)** Densitometric analysis of CD206 expression. **(H)** Bar graph showing the ratio analysis of p-PI3K/PI3K. **(I,J)** ELISAs performed for the TNF-α and IL-1β in culture medium obtained at 24h after LPS stimuli with 0.2 μg/mL and 1μg /mL Ex-4 treatment or not (n=6). The error bars represent the SD. *p < 0.05 vs. control group, #p < 0.05 vs. LPS group by one-way ANOVA followed by Tukey's post hoc analysis (*p<0.05, **p<0.01, and ***p<0.001).

To identify the specific cells expressing GLP-1R, the cords of control mice were extracted, followed by IF staining. Fig. 1A shows that the GLP-1R largely overlapped with NeuN and IBA-1; however, the GFAP positive cells were rarely overlaid with GLP-1R. We therefore determined whether the agonistic action of GLP-1R in microglia could mitigate neurological insults following SCI. The dose of Ex-4 treatment was preliminarily determined using western blotting (Figure S2A-B). The results showed that the concentrations of Ex-4 of 0.2-1 μg/mL reduced iNOS levels in microglia. The further western blot results showed that LPS treatment significantly increased the expression of iNOS, while a significant decrease was shown in microglia treated with Ex-4 at 1 μg/mL (Figure 1B-C). Additionally, the increased expression of cyclooxygenase-2 (COX-2) after LPS treatment was also decreased by Ex-4 treatment at doses from 0.2 μg/mL to 1 μg/mL (Figure 1B, D). However, the expression of GLP-1R was not affected by LPS and Ex-4 treatments (Figure 1B,E). Notably, the expression of the M2 subtype of CD206 of microglia was decreased after LPS treatment, whereas Ex-4 treatments at doses of 1 μg/mL significantly reversed the low expression of CD206 in microglia treated with LPS (Figure 1F-G). In addition, Ex-4 treatment increased the level of PI3K phosphorylation in inflammatory microglia (Figure 1F-H). The representative proinflammatory cytokines were measured using ELISAs, showing that the high expressions of TNF-α and IL-1β in microglial media after LPS treatment were significantly reduced after treatments with 0.5 μg/mL and 1 μg/mL of Ex-4, respectively (Figure 1I-J).

### GLP-1R Activation Modulates Polygenic Expression in Activated Microglia

**Figure 2:**
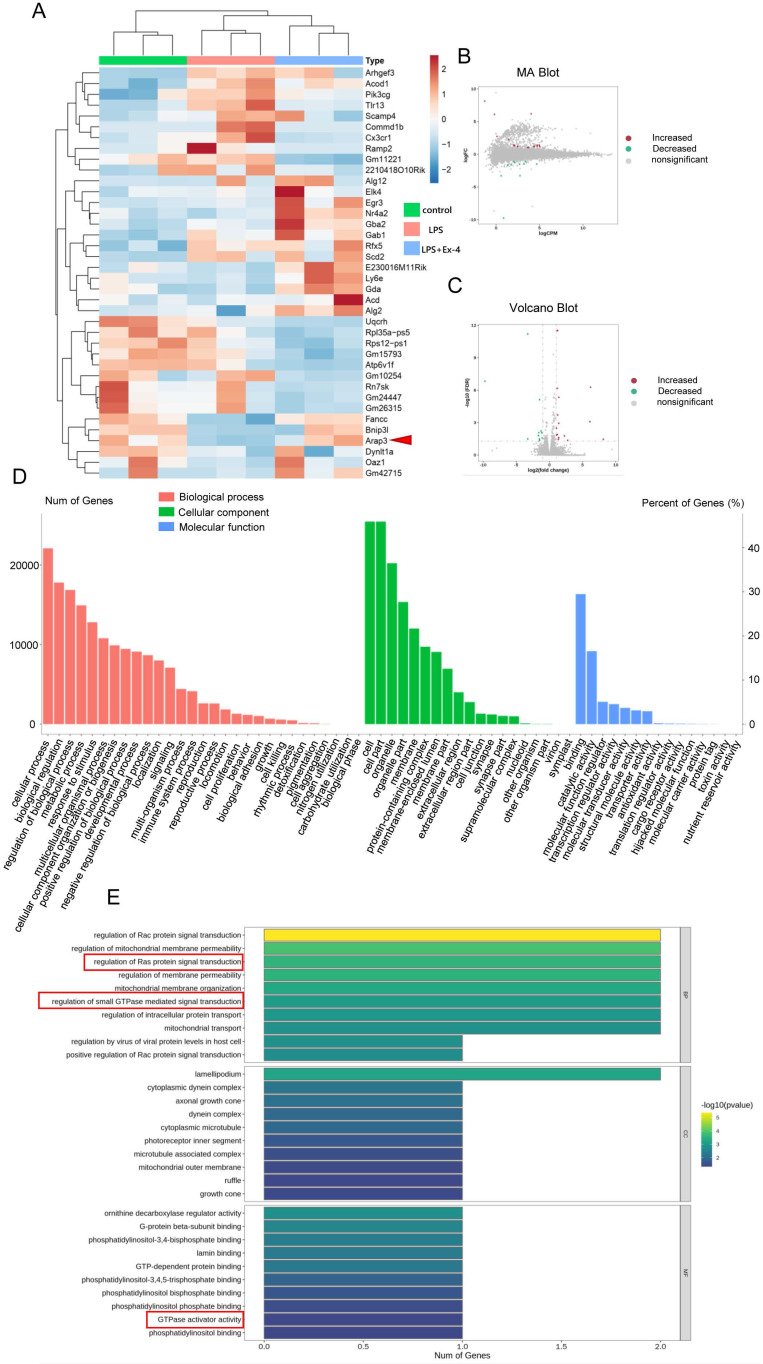
** GLP-1R activation modulates polygenic expression in activated microglia. (A)** A heat map showed differentially expressed genes (DEGs) in LPS activated microglia that were pretreated with Ex-4 or not. **(B)** MA diagram provided an intuitive view of DEGs distribution and differential multiples in LPS group and LPS+Ex-4 group. **(C)** Volcano Plot showed DEGs in LPS group and LPS+Ex-4 group. **(D-E)** Based on DEGs and all genes, the gene enrichment of GO and KEGG analysis reflected the status of the secondary functions in the three backgrounds and Ras signaling with GTPase was involved (marked by red frame).

RNA-seq was further used to identify the mechanisms of GLP-1R activation during microglial inflammation. Analysis of RNA-seq exhibited remarkable alterations in mRNA after 1 μg/mL Ex-4 and LPS treatments (Figure 2A). The transcriptome investigation showed 38 differentially expressed genes (DEGs) in the three groups, containing 16 increased and 11 decreased genes (Figure 2B-C). GO annotation showed that the enrichments of DEGs were mostly distributed in multiple biological processes, cellular components, and molecular functions (Figure 2D). Interestingly, we found that the trends of DEGs participated in regulation of Ras protein signal transduction, regulation of small GTPase-mediated signal transduction, and GTPase activator activity by using the KEGG database (Figure 2E). Moreover, ARAP3 expression was found to be significantly altered with iNOS expression post-GLP-1R activation by different concentrations of Ex-4 (Figure S2A-B), but the systemic regulatory mechanism of microglial inflammatory interference by GLP-1R activation requires further verification.

### GLP-1R Activation Amends Microglial Inflammatory Cascades via Modulation of the GTPase Activating Protein, ARAP3

**Figure 3:**
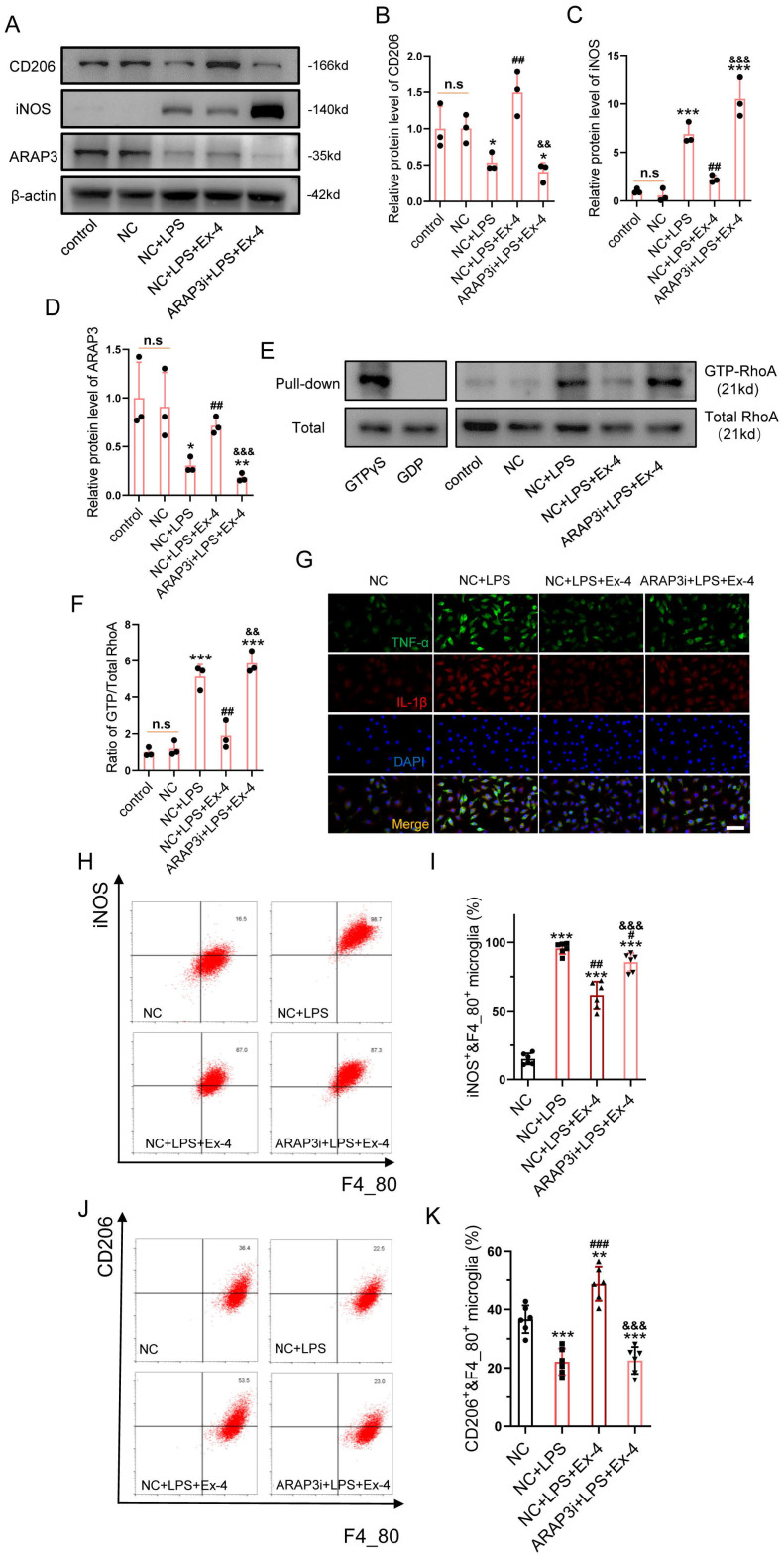
** GLP-1R activation amends microglial inflammatory cascades via modulation of the GTPase activating protein ARAP3. (A)** Western blotting performed for the proteins including CD206, iNOS, ARAP3 in LPS-activated microglia treated with Ex-4 after transfection of ARAP3 interference (ARAP3i) or NC; n=3. β-actin was used as the control. **(B)** Densitometric analysis of CD206 expression. **(C)** Bar graph showing the densitometry analysis of iNOS expression.** (D)** Densitometric analysis of ARAP3. **(E)** Western blotting for GTP-RhoA and Total RhoA in LPS-activated microglia treated with Ex-4 after transfection of ARAP3 interference (ARAP3i) or NC; n=3. **(F)** Bar graph showing the ratio analysis of GTP-RhoA/Total RhoA.** (G)** Representative immunofluorescence labeling images for TNF-α (green) and IL-1β (red) in LPS-activated microglia treated with Ex-4 after transfection of ARAP3 interference (ARAP3i) or NC; Scale bar = 50 μm.** (H-I)** Representative flow cytometry performed for the distinction of CD11c^+^&F4_80^+^ and CD206^+^&F4_80^+^ microglia transfected ARAP3i or NC and treated with Ex-4 post 12h LPS inducement.** (J-K)** Bar graph showing the quantitative analysis of the percent; n=6. The error bars represent the SD. *p < 0.05 vs. NC group, #p < 0.05 vs. NC+LPS group, &p vs. NC+LPS+Ex-4 group by one-way ANOVA followed by Tukey's post hoc analysis (*p<0.05, **p<0.01, and ***p<0.001).

The specific target, ARAP3 in GLP-1R activation-induced anti-inflammation was investigated. After cells were transfected with GFP-labelled shRNA, we ascertained an optimal LV titer for knockdown of ARAP3 using fluorescence assays (Figure S3). Western blotting further showed that decreased iNOS expression and increased CD206 expression were reversed after Ex-4 treatment in LPS-stimulated microglia^ARAP3i^ (Figure 3A-C). The expression of ARAP3 was decreased in microglia^ARAP3i^, when compared with NC microglia (Figure 3A, D). The RhoA activation assay showed that Ex-4 treatment significantly reduced the level of GTP-RhoA after LPS inducement in NC microglia; however, its function was not present in microglia^ARAP3i^ (Figure 3E-F). Moreover, the inflammatory cytokines, TNF-α and IL-1β, were measured using IF, which showed that the decreased levels of TNF-α and IL-1β in Ex-4-treated microglia were reversed by ARAP3i transfection (Figure 3G). The polarization of microglia was determined using flow cytometry, which showed that LPS treatment increased the relative percentages of CD11c^+^ and F4_80^+^ cells, while it reduced that of CD206^+^&F4_80^+^ cells, and GLP-1R agonism significantly reduced the percentage of iNOS^+^&F4_80^+^ glial cells and increased the percentage of CD206^+^&F4_80^+^ microglia. Consistently, the corrective effect of GLP-1R activation on the polarization of the microglial subtype was reversed by ARAP3i transfection (Figure 3H-J).

### Neuroinflammation-induced Astrogliosis Is Mitigated by Agonist Treatment of GLP-1R in Microglia

**Figure 4:**
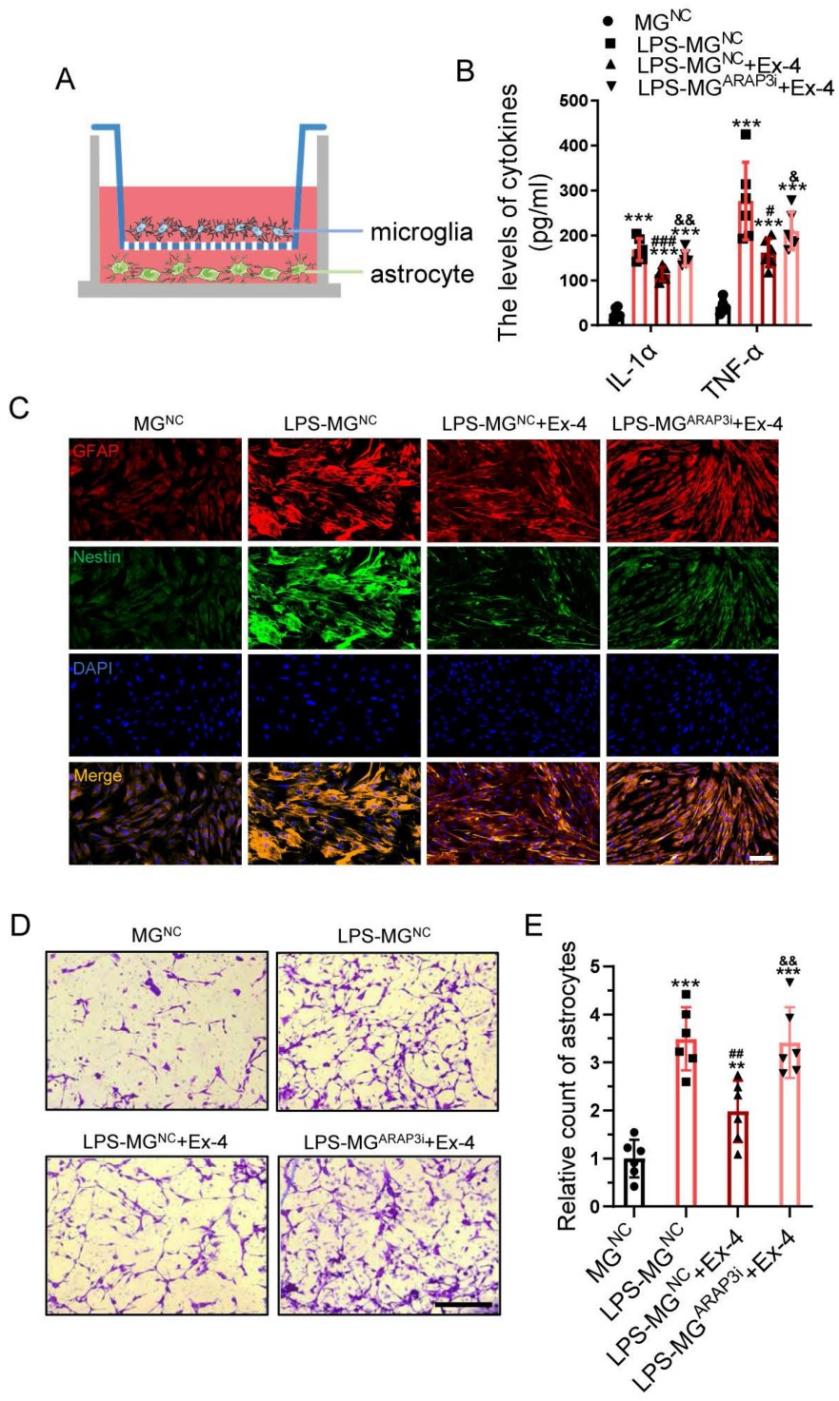
**Neuroinflammation-induced astrogliosis is mitigated by agonist treatment of GLP-1R in microglia. (A)** Diagrammatize method of co-culture between primary microglia and astrocytes. **(B)** ELISAs performed for the TNF-α and IL-1α in culture medium obtained at 24h after LPS stimuli with Ex-4 treatment or not (n=6). **(C)** Representative immunofluorescence labeling images for Nestin (green) and GFAP (red) obtained from primary astrocyte co-cultured with microglia in MG^NC^, LPS-MG^NC^, LPS-MG^NC^+Ex-4, and LPS-MG^ARAP3i^+Ex-4 group for 48h, respectively. The blue staining indicates the DAPI-stained nuclei. Scale bar = 50 μm. **(D)** Representative images of astrocytes stained with crystal violet in trans-well assay, which were treated with microglial medium in MG^NC^, LPS-MG^NC^, LPS-MG^NC^+Ex-4, and LPS-MG^ARAP3i^+Ex-4 group for 48h. **(E)** Quantitative analysis of the amount of astrocytes under inserts; Scale bar = 100 μm, n=6. The error bars represent the SD. *p < 0.05 vs. MG^NC^ group, #p < 0.05 vs. LPS-MG^NC^ group, and &p< 0.05 vs. LPS-MG^NC^+Ex-4 by one-way ANOVA followed by Tukey's post hoc analysis (*p<0.05, **p<0.01, and ***p<0.001).

We next conducted co-culturing of microglia and astrocyte as shown in Figure 4A. The primary functional cytokines generated by microglia, such as TNF-α and IL-1α, were determined in the culture medium in each group, showing LPS-treated negative control microglia (LPS-MG^NC^) released some promoters of astrocyte activation, when compared with the negative control microglia (MG^NC^) group, whereas microglia treated with LPS and Ex-4 exhibited a significantly decreased cytokine level. Notably, the ARAP3-interfered microglia (MG^ARAP3i^) reversed the Ex-4-induced cytokines decrease after LPS treatment (Figure 4B). The activation of reactive astrocytes was further visualized using IF staining, showing that astrocytes co-cultured with LPS-MG^NC^ significantly expressed more GFAP and Nestin and possessed larger bodies than those co-cultured with MG^NC^. However, the reactive astrocytes co-cultured with LPS-MG^NC^ exhibited a dramatic reduction, both of the GFAP/Nestin expression and cell bodies following Ex-4 treatment, which was increased in LPS-MG^ARAP3i^ treated with Ex-4 (Figure 4C). The migration of astrocytes was also measured using Transwell assays (Figure 4D). We found that LPS-MG medium increased the migration of astrocytes, while the number of cells that migrated through the hole was significantly reduced in the LPS-MG^NC^+Ex-4 group. Interestingly, MG^ARAP3i^ minimally but significantly reduced the migration of astrocytes post-Ex-4 treatment (Figure 4E).

### Administration of the GLP-1R Agonist Alleviates Neuroinflammation by Up-regulation of ARAP3 at the Acute Stage of SCI

**Figure 5:**
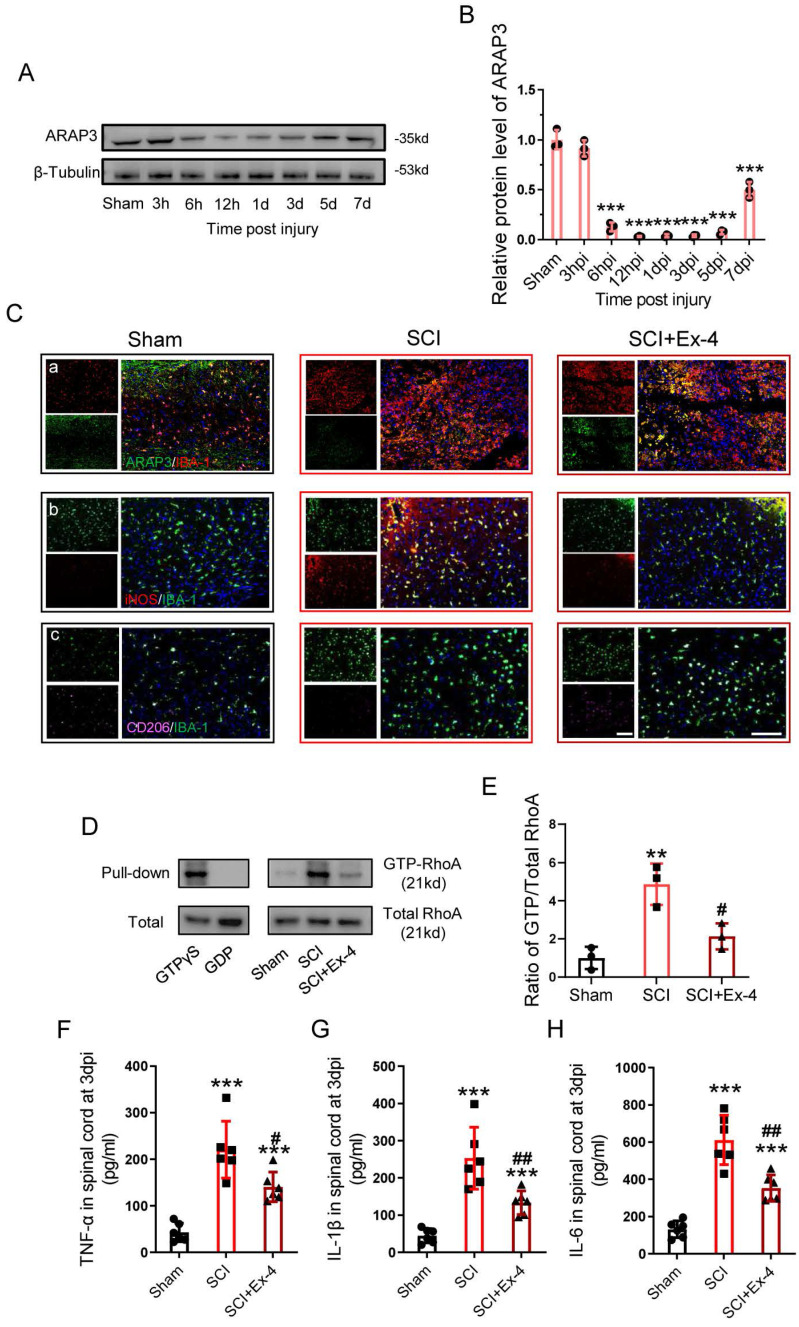
** Administration of the GLP-1R agonist alleviates neuroinflammation at the acute stage of SCI. (A)** Western blotting for ARAP3 expression within 1 week post SCI. **(B)** Bar graph showing a quantitative analysis of ARAP3 expression; n=3. **(C)** Double immunofluorescence labeling of microglia for (a) IBA-1(red)/ARAP3(green), (b) IBA-1 (green)/iNOS (red), and (c) IBA-1 (green)/CD206 (pink) obtained from longitudinal sections centered around central canal at 3 dpi in Sham, SCI and Ex-treated SCI mice. As shown in the Ex-treated mice, iNOS was down-regulated but CD206 was increased in the microglia. Scale bar = 100 μm. **(D)** Western blots performed for the GTP-RhoA and Total-RhoA in tissues obtained at 3 dpi after SCI.** (E)** Bar graph showing a quantitative analysis of the ratio of GTP-RhoA/Total-RhoA; n=3.** (F-H)** ELISAs performed for the TNF-α, IL-1β, and IL-6 expressions in injured tissue obtained at 3 dpi, showing significantly decreased levels of TNF-α, IL-1β, and IL-6 in SCI+Ex-4 group; n=6. The error bars represent the SD. *p < 0.05 vs. Sham group, #p < 0.05 vs. SCI group by one-way ANOVA followed by Tukey's post hoc analysis (*p<0.05, **p<0.01, and ***p<0.001).

The expression of ARAP3 was examined within a week post-SCI, showing that ARAP3 expression was reduced at 6 h post-injury (hpi), but remained at a significantly low level for 12 hpi to 5 dpi, and increased at 7 dpi (Figure 5A-B).

At 3 dpi, the sections centered in the injured cords were analyzed using double staining (ARAP3/IBA-1, iNOS/IBA-1 or CD206/IBA-1), showing that ARAP3 expression was low in microglia of injured cords, whereas its level increased under Ex-4 treatment (Figure 5Ca); SCI induced microglia activation in the M1 subtype, involving expression of iNOS, while the M2 subtype expressing CD206 was rarely observed; however, injured cord treated with Ex-4 showed reduced iNOS positive microglia and increased CD206 positive microglia, when compared with untreated cords (Figure 5Cb-Cc). Furthermore, we measured the activation of RhoA in injured cords, which showed that the level of GTP-RhoA was increased following SCI. Importantly, Ex-4 treatment led to a decreased level of GTP-RhoA, when compared with the SCI group (Figure 5D-E). Additionally, the levels of TNF-α, IL-1β, and IL-6 were measured at 3 dpi, showing a significant elevation of TNF-α, IL-1β, and IL-6 levels; but administration of GLP-1R agonist significantly reduced the expressions of these proinflammatory cytokines (Figure 5F-H).

### Agonist Treatment of GLP-1R in Spinal Cord Corrects Glial Assembly and Prevents Secondary Neurological Damage Post-SCI

**Figure 6:**
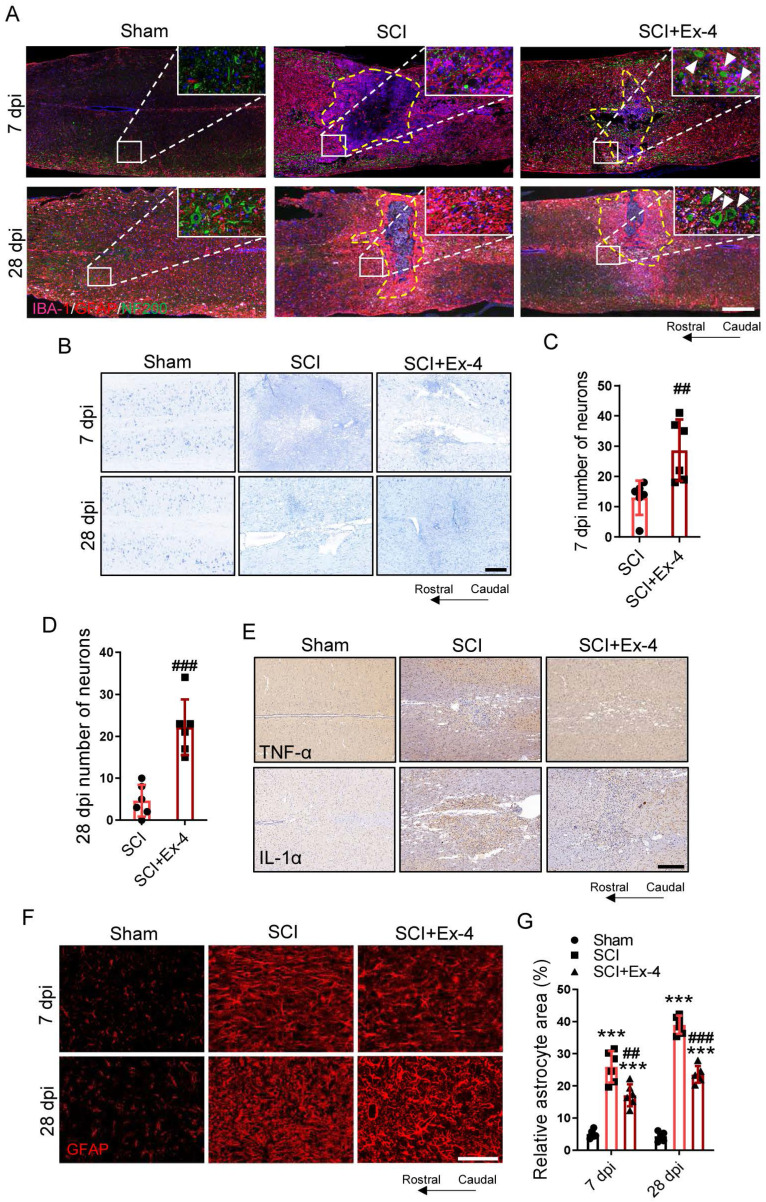
** Agonist treatment of GLP-1R in spinal cord corrects glial assembly and prevents secondary neurological damage post SCI. (A)** Triple immunofluorescence labeling of microglia for IBA-1 (pink), astrocytes for GFAP (red) and neurons and neurofilaments for NF-200 (green) obtained from longitudinal sections centered around the injured core 3 mm at 7 dpi and 28 dpi in Sham, SCI and Ex-treated SCI mice; Scale bar = 500 μm. **(B)** Representative images for Nissl staining obtained from longitudinal sections centered around the injured core 1.5 mm at 7 dpi and 28 dpi in Sham, SCI and Ex-treated SCI mice. Scale bar = 250 μm. **(C)** Quantitative analysis of the amount of survived neurons at 7 dpi; n=6. **(D)** Quantitative analysis of the amount of survived neurons at 28 dpi; n=6. **(E)** Representative immunohistochemistry labeling images for TNF-α and IL-1α expressions obtained from longitudinal sections centered around the injured core 1.5 mm at 3 dpi in sham, SCI and Ex-treated SCI mice. Scale bar = 250 μm. **(F)** Representative immunofluorescence labeling of astrocytes for GFAP (red) obtained from longitudinal sections 1 mm caudal to the lesion site at 7 dpi and 28 dpi. Scale bar = 100 μm. **(G)** Quantitative analysis of the area of astrocyte scar at 7dpi and 28 dpi; n=6. The error bars represent the SD. *p < 0.05 vs. Sham group, #p < 0.05 vs. SCI group by one-way ANOVA followed by t-test and Tukey's post hoc analysis (*p<0.05, **p<0.01, and ***p<0.001).

At 7 and 28 dpi, the structural distribution of primary cells including neurons, astrocytes, and microglia in the injured cords were determined using IF triple staining. The images shown in the larger injured area had more glial scarring as well as fewer neurons and neurofilaments surrounding the injured foci in the SCI group, when compared with those in the SCI+Ex-4 group (Figure 6A). Nissl staining was performed at 7 and 28 dpi. The results showed that the number of surviving neurons in mice treated with Ex-4 was significantly more than that in untreated mice, when assessed at the same magnification (Figure 6B-D). IHC staining showed that the expressions of TNF-α and IL-1α near the injured foci increased following trauma, while Ex-4 treatment reduced the levels of TNF-α and IL-1α surrounding the injury cord (Figure 6E). The areas of reactive astrocytes adjacent to the injured cord at 7 and 28 dpi were revealed by IF staining, showing that the percentage of astrocyte positive areas was significantly increased, but decreased significantly after administration of Ex-4 at 7 and 28 dpi (Figure 6 F-G).

### The Potential Role of the GLP-1R Agonist in Myelin Protection Following SCI

**Figure 7:**
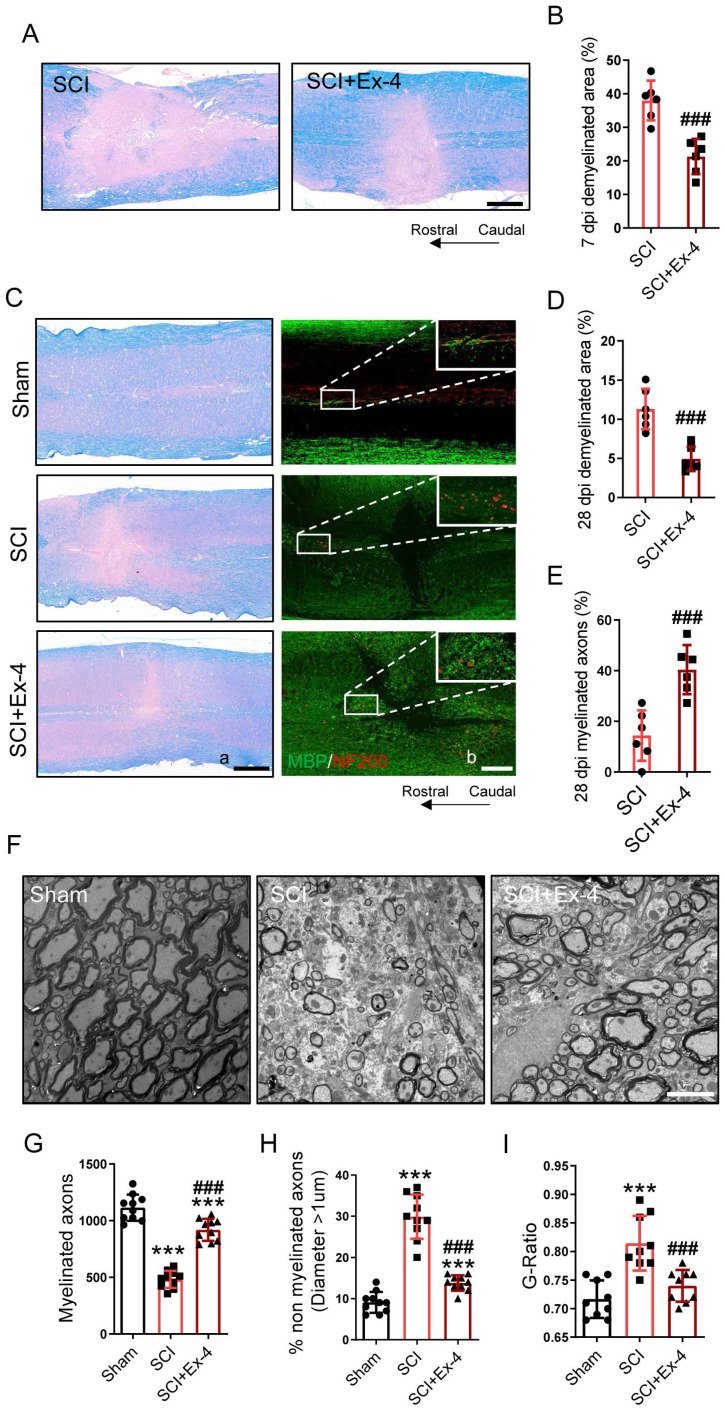
** The potential role of the GLP-1R agonist in myelin protection following SCI. (A)** Representative images for LFB staining obtained from longitudinal sections centered around the injured core 3 mm at 7 dpi in SCI and Ex-treated SCI mice; Scale bar = 500 μm.** (B)** Quantitative analysis of the demyelinated area at 7dpi; n=6.** (C) (a)** Representative images for LFB staining obtained from longitudinal sections centered around the injured core 3 mm at 28 dpi in Sham, SCI and Ex-treated SCI mice; Scale bar = 500 μm; **(b)** Representative immunofluorescence labeling of myelin sheath for MBP (green) and neurofilaments for NF-200 (red) obtained from longitudinal sections centered around the injured core 1.5 mm at 28 dpi; Scale bar = 250 μm. **(D-E)** Quantitative analysis of the demyelinated area and remyelinated axons at 28 dpi; n=6.** (F)** Representative EMI images for myelin sheath and neurofilaments 1 mm caudal to the lesion site at 28 dpi; Scale bar = 5 μm. **(G)** Quantitative analysis of the amount of myelinated axons; n=9.** (H)** Quantitative analysis of the amount of non-myelinated axons whose diameter more than 1 μm; n=9. **(I)** Quantitative analysis of G-ratio; n=9. The error bars represent the SD. *p < 0.05 vs. Sham group, #p < 0.05 vs. SCI group by one-way ANOVA followed by Tukey's post hoc analysis (*p<0.05, **p<0.01, and ***p<0.001). Quantitative analysis of the area of astrocyte scar at 7dpi and 28 dpi. The error bars represent the SD. *p < 0.05 vs. Sham group, #p < 0.05 vs. SCI group by one-way ANOVA followed by t-test and Tukey's post hoc analysis (*p<0.05, **p<0.01, and ***p<0.001).

Demyelination in the injured cords was shown using LFB staining at 7 dpi. The results showed that SCI induced excessive demyelinated regions centered in the injured cord, whereas administration of GLP-1R agonist significantly reduced the demyelinated areas in the cords, when compared with those in the SCI group (Figure 7A-B). In addition, the level of remyelination at the late period of SCI was detected by LFB and IF staining. At 28 dpi, the non-myelinated areas in the injured cords significantly decreased in mice treated with Ex-4. Besides, SCI caused extensive NF200-labeled axons without a covering of myelin basic protein (MBP), but Ex-4 treatment increased the MBP-coated axons surrounding the injured foci (Figure 7C-E). Moreover, the EMI results showed a drastic reduction of myelinated axons and a significant increase of non-myelinated axons in the area neighboring the injured cord, while the increased myelinated axons and reduced non-myelinated axons were found in the SCI+Ex-4 group at 28 dpi (Figure 7F-H). Further analysis also showed a reduced G-Ratio of axons following Ex-4 treatment (Figure 7I).

### GLP-1R Agonist Reduces Tissue Destruction and Locomotor Dysfunction in SCI Mice

**Figure 8:**
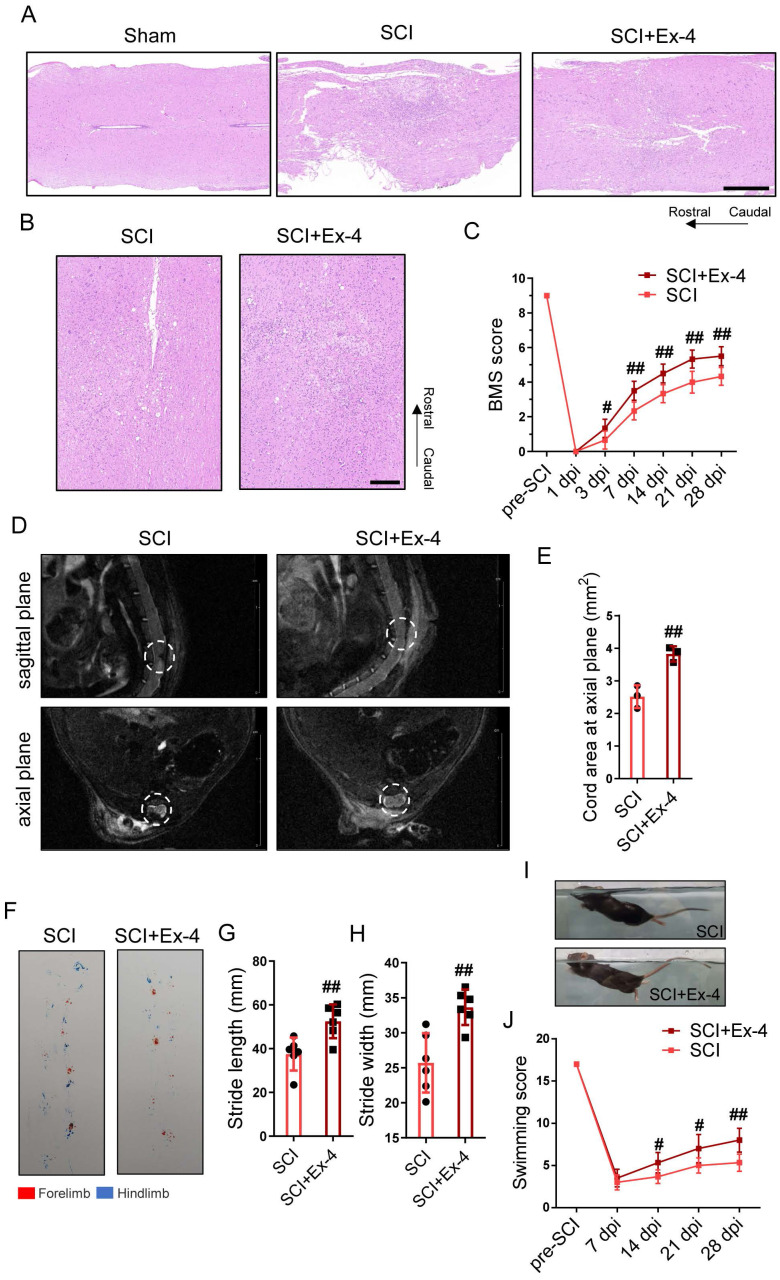
** The glucagon-like peptide-1 receptor agonist reduces tissue destruction and locomotor dysfunction in spinal cord injury (SCI) mice. (A)** Longitudinal sections of cords centered 3 mm around the injured core obtained at 7 dpi as indicated. The cords were stained with hematoxylin and eosin (HE). Evidence of tissue loss was observed in the sections; Scale bar = 500 μm. **(B)** HE staining images of cords centered 1.5 mm around the injured core obtained at 28 dpi; Scale bar = 200 μm. **(C)** Locomotor recovery of hind limbs was assessed according to the BMS open-field test, n=6. **(D)** Representative magnetic resonance images in injured foci of cords at the sagittal plane and axial plane at 28 dpi. **(E)** Quantitative analysis of spinal cord areas at the axial planes in mice; n=3. **(F)** A footprint analysis performed at 28 dpi showed that Ex-4 treated mice were able to take more consistent steps. **(G-H)** Quantification of the footprint analysis after SCI showing significantly better functional recovery in Ex-4 treated mice than in SCI mice at 28 dpi; n=6. **(I-J)** Photos of swimming at 28 dpi, showing the worse trunk instability and uncoordinated action in SCI mice, and statistical analysis of the Louisville Swim Scale over a 28 day period; n=6. ^*^p < 0.05 vs. the Sham group, ^#^p < 0.05 vs. the SCI group using the *t*-test, one-way analysis of variance followed by Tukey's post hoc analysis (^*^p<0.05, ^**^p<0.01, and ^***^p<0.001).

To analyze the neurohistology following trauma, injured cords with or without Ex-4 treatment were measured post-SCI using HE staining. The results showed that the injured area of cords undergoing GLP-1 agonist treatment was smaller than that of the untreated cords at 3, 7 and 28 dpi (Figure S4B). The HE staining at 7 dpi exhibited larger tissue loss and more cellular infiltration in the SCI group than those in the SCI+Ex-4 group (Figure 8A), and staining at 28 dpi consistently showed that the untreated cords contained more histological defects and a broader injured cord than the Ex-4-treated cords (Figure 8B). Moreover, the mi-MRI in the sagittal plane and axial plane showed that the defective tissue areas in the Ex-4-treated mice were decreased, when compared with that in the untreated mice at 28 dpi (Figure 8D-E). The result of the BMS score test showed that the score of the SCI+Ex-4 group was significantly higher than that of the SCI group, beginning at 3 dpi and remaining at 28 dpi (Figure 8C). Footprint assays at 28 dpi showed higher stride length and width as well as more coordinated gait in the SCI+Ex-4 group, when compared with the SCI group (Figure 8F-H). Regarding the swimming test, the results showed that the 28 dpi mice treated with Ex-4 occasionally swam with their hind limbs swinging and tails hanging on the water to keep balance. Importantly, the angle between the back plane and the water surface in the SCI+Ex-4 group was smaller than that in the SCI group (Figure 8I). Hence, the higher swimming score in the SCI+Ex-4 group beginning at 14 dpi was more significant at 28 dpi (Figure 8J).

## Discussion

**Figure 9:**
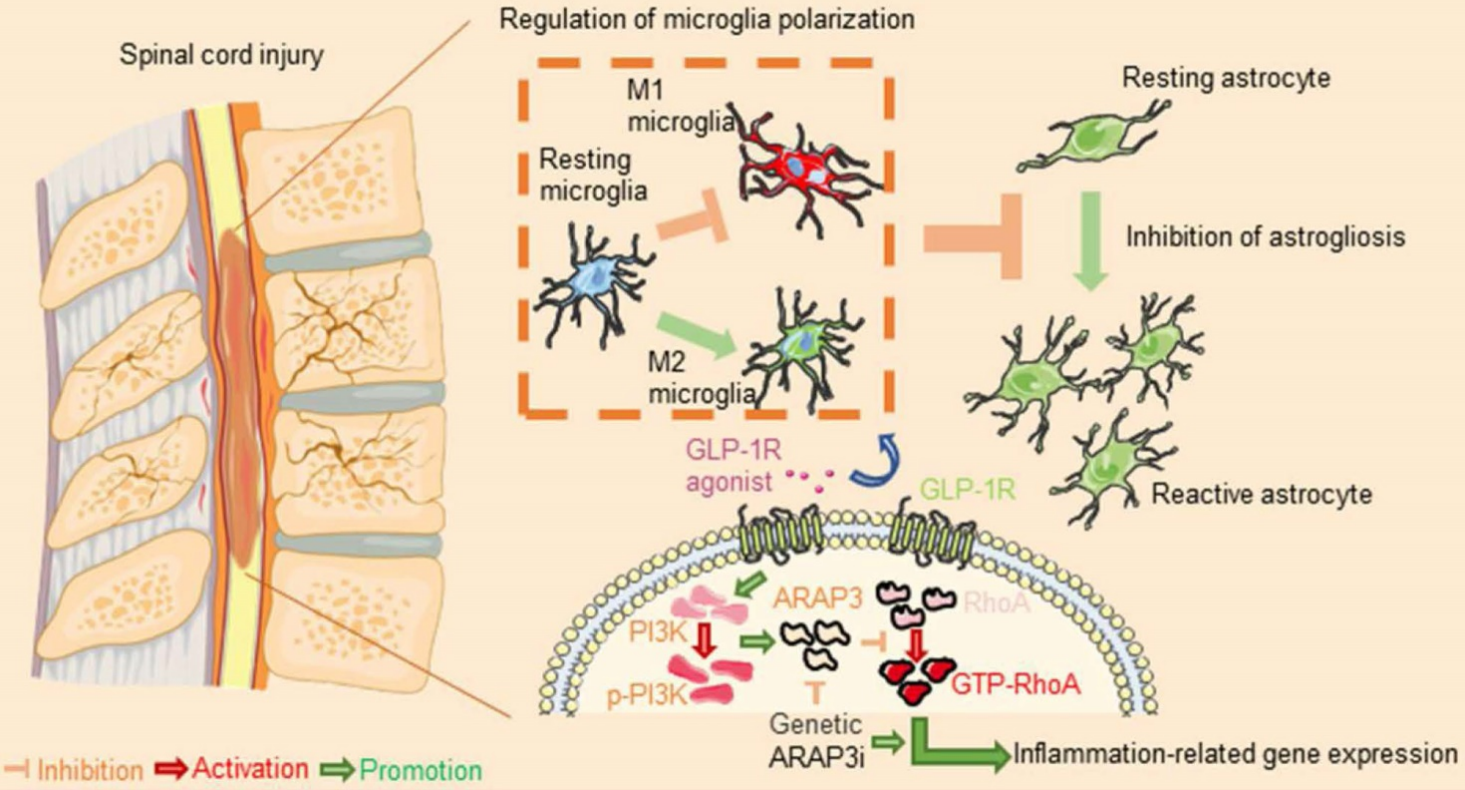
After spinal cord injury, resident microglia differentiated into M1 subtype cells, resulting in neuroinflammation, followed by astrogliosis. However, glucagon-like peptide-1 receptor (GLP-1R) activation reversed microglial polarization to the M2 subtype to attenuate inflammatory processes. Mechanically, the GLP-1R agonist rescued ARAP3 expression by increasing its upstream PI3K phosphorylation and further inhibited RhoA activation. Inhibition of ARAP3 showed a significant antagonistic effect on GLP-1R activation-induced anti-inflammation.

Our results suggested that the ameliorative effects of Ex-4 treatment could be modulated in part by the mechanism shown in **Figure 9** and described as follows. First, the regulation of microglial activation via the PI3KARAP3/RhoA axis attenuated the degree of neuroinflammatory response-induced secondary injury. Second, the decreased reactive astrocyte-driven cytokines secreted by microglia mitigated the formation of glial scar post SCI.

A previous study has reported that GLP-1R in mouse spinal cord was largely located in microglia cells with a small amount in neurons and astrocytes 31. However, we confirmed that GLP-1R was not only widely expressed in microglia, but was also expressed in neurons. Importantly, we rarely found the expression of GLP-1R in astrocytes of the spinal cord, which potentially meant that GLP-1R activation did not directly affect the biological activity of astrocytes following SCI. Administration of GLP-1R agonist inhibited neuronal apoptosis, regulated autophagy, and promoted axonal regeneration after SCI 32,33. Considering that the regulatory role of GLP-1R agonism in neurons has been reported, we herein revealed the potential role and mechanism of GLP-1R in microglia and astrocytes that may be interactively affected after SCI. Following SCI, the inducement of microglia is one of the main forces causing neuroinflammatory responses. Microglia respond to injury stress by releasing a variety of proinflammatory mediators 2,34,35 and they work with astrocytes to form a barrier to block aggravating secondary injury resulting from the diffusion of necrotic substances in the injured cord 36. Unfortunately, the excessive inflammatory phenotype of microglia exacerbates the development of secondary injury 37, and recruits the collective glial population to form larger areas of glial scar surrounding the lesion, which further hinders axonal regeneration and neurological remodeling 1,33,38. GLP-1R activation in microglia has been shown to have a palliative effect on neuroinflammation in various neurological diseases 39-41. We similarly treated LPS-mediated inflammatory microglia with GLP-1R agonist in different dose gradients *in vitro*, and found that the GLP-1R agonist at a dose of 1 μg/mL reduced the expressions of inflammatory mediators like iNOS and COX-2 as well as multiple proinflammatory factors including TNF-α, IL-1β, and IL-6. Microglial polarization plays a bidirectional regulatory role in neuroinflammatory regulation 42,43. M1 type differentiation dominates the inflammation of microglia, while M2-type polarization antagonizes the aggravation of inflammation 44,45. In a spinal cord neurogenic pain model, GLP-1R activation of microglia was found to increase the expression of M2 subtype markers rather than M1 subtype markers 22. Consistently, we detected the double polarization markers of microglia after GLP-1R agonist treatment, showing that reduced iNOS levels were accompanied by increased CD206 expression. These findings suggested that microglial GLP-1R may regulate the development of neuroinflammation through microglial polarization after SCI.

To more clearly understand the mechanism by which GLP-1R agonist modulates polarization in microglia cells, we further employed the RNA-seq technique to show polygenic differences among the resting microglia and the activated microglia with or without Ex-4 treatment. The analysis showed that a decrease of ARAP3 expression was found in activated microglia, and its expression was restored post-Ex-4 administration, indicating that ARAP3 was partly involved in the anti-inflammation process regulated by GLP-1R. Using LV-shRNA-ARAP3 interference and pharmacological activation of GLP-1R, we traced the action between ARAP3 protein and GTP-RhoA synthesis, and identified the mechanism by which microglia induced neuroinflammation. Several studies 46-48 have reported that activation of the RhoA signaling pathway was involved in the microglial inflammatory response in numerous diseases. Importantly, abundant expression of ARAP3 has been shown to play a negative role in RhoA activation 49-51. Our results showed that an GLP-1R agonist significantly increased the transcription and translation levels of ARAP3 in inflammation-activated microglia, and inhibited activation of the downstream target, RhoA, whereas the effect was antagonized by genetic knockdown of ARAP3. It is suggested that GLP-1R activation mitigates the level of neuroinflammation partly by increasing the expression of ARAP3 and inhibiting the phosphorylation of GDP-RhoA in microglia. ARAP3 is a type of PI3K- and Rap-modulated GTPase activating protein for RhoA, and is involved in the inflammatory process of cells 49,51,52. Our *in vitro* results not only adequately supported ARAP3 rescue regulating microglia polarization during neuroinflammation, but also complemented the potential signaling pathway of GLP-1R agonist in reducing the level of inflammation. Yun and co-workers showed that activation of GLP-1R in microglia effectively blocked the conversion of toxic astrocytes in Parkinson's disease 53. Moreover, the release of various cytokines secreted by microglia, including IL-1α, TNF-α and C1q, induced the activation of resting astrocytes into reactive astrocytes 54-56. We also found that astrocytes co-cultured with the GLP-1R agonist-treated microglia became insensitive, and were accompanied by reduced migration. Additional results showed that the secretion of astrocyte-promoting activators like IL-1α and TNF-α by microglia into the culture medium was significantly reduced, suggesting that GLP-1R agonist treatment may hinder the inflammation-mediated glial effects.

At 3, 7, and 28 dpi, we assessed the neuroinflammatory level, glial scar formation, and neural structural damage in models of SCI. First, microglial polarization characterized by significantly reduced M1 phenotypes and increased M2 phenotypes in injured cords after EX-4 treatment were found to be consistent with changes *in vitro*. Importantly, the elevated expression of ARAP3 and decreased activation of RhoA were found in Ex-4-treated mice. Significant decreases in proinflammatory factors including TNF-α, IL-1β, and IL-6 were detected in injured cord tissue after Ex-4 treatment. The level of neuroinflammation was positively correlated with secondary SCI. Longer durations and greater outbreaks of neuroinflammation led to more deterioration adjacent to the primary injury, such as more neuron loss, worse demyelination, and larger dispersive glial scarring 57-59. Therefore, therapy to restrict neuroinflammation is critical for the rescue of more neurological histology. In the current study, the results showed fewer glial populations and more surviving neurons in the GLP-1R agonist-treated spinal cord tissue surrounding the injured cord, suggesting that the administration of GLP-1R agonist may potentially improve the microenvironment of neurons by reducing neuroinflammation. It has been reported that the expression of astrocytes peaks at 7 dpi and then gradually decreases and stabilizes 60,61. However, we showed that the astrocyte positive area at 28 dpi was slightly more than that at 7 dpi, which may be the consequence of cell hypertrophy. The decreased distribution and level of reactive astrocyte promoters adjacent to the injured foci after Ex-4 treatment partly explained the reduced astrocyte scar area around the injured epicenter at 7 dpi and 28 dpi. Oligodendrocytes are the main components of the myelin sheath, and play an irreplaceable role in the stability and signal transduction of neural functional units 62,63. Neuroinflammation with activated inflammatory microglia post-SCI is closely related to the excessive death of oligodendrocytes and extensive axonal retraction 64-66. Inflammatory inhibition in microglia has been shown to be effective in reducing demyelination in an experimental autoimmune encephalomyelitis model 67. We confirmed that extensive demyelination in the spinal cord centered around the injured cord at 7 dpi, while Ex-4 treatment attenuated the development of demyelination to a large extent. Subsequently, myelinated axons were significantly increased in mice treated with the GLP-1R agonist, when compared with the untreated mice at 28 dpi. The overall improvements in neurohistology may therefore be closely associated with the recovery of hind limb locomotor function.

There were a few limitations that should be recognized. First, we evaluated the effect of GLP-1R agonist on the expression of related factors in neuroinflammation using silencing of ARAP3 in microglia *in vitro*, but conditioned knockout of ARAP3 in microglia *in vivo* would be more revealing. Second, we only assessed the representative drivers secreted by microglia to astrocytes, whereas we may have overlooked some other microglial cytokines affected by the GLP-1R agonist. Third, although we focused on the protective effect of Ex-4-mediated neuroinflammatory inhibition on myelin sheath *in vivo*, our study failed to distinguish whether the observations were the direct effect of GLP-1R agonist on oligodendrocytes or indirect regulation through interactions of microglia and oligodendrocytes. Finally, an observed side effect of Ex-4 administration was significant weight loss in mice, which may be a functional problem to be resolved in the treatment of clinical patients. Nevertheless, this study demonstrated the inhibitory effect of GLP-1R on microglia-mediated neuroinflammation post-SCI. GLP-1R agonists may improve the neurological function of patients, especially those with diabetes, by regulating the polarization of microglia. In summary, agonistic action of GLP-1R by Ex-4 exerted a neuroprotective effect in SCI by attenuating inflammation-induced microglia-astrocytes interactions and histological breakdown in a microglia-dependent manner. Mechanistically, GLP-1R agonist inactivating RhoA signaling may regulate microglial polarization through rescue of ARAP3 expression. The GLP-1R agonist thus represents a promising drug for treatment of traumatic and inflammatory nerve injury.

## Supplementary Material

Supplementary figures.

## Abbreviations

ARAP3: Arf and Rho GAP adapter protein 3
ARAP3i: ARAP3 interference
BMS: Basso Mouse Scale
COX-2: Cyclooxygenase-2
DAPI: Diaminobezidin
DMEM: Dulbecco's Modified Eagle Medium
DEGs: Differential expressed genes
dpi: day post-injury
ELISA: Enzyme-linked immunosorbent assay
EMI: Electron microscope imaging
Ex-4: Exendin-4
FBS: Fetal bovine serum
GAPDH: Glyceraldehye 3-phosphate dehydrogenase
GFAP: Glial fibrillary acidic protein
GLP-1R: Glucagon-like peptide-1 receptor
GO: Gene oncology
HE: Hematoxylin-eosin
IBA-1: Ionized calcium binding adaptor molecule 1
IF: Immunofluorescence
IHC: Immunohistochemistry
IL-1α: Interleukin-1α
IL-1β: Interleukin-1β
IL-6: Interleukin-6
iNOS: Inducible nitric oxide synthase
KEGG: Kyoto Encyclopedia of Genes and Genomes
LFB: Luxol Fast Blue
LPS: Lipopolysaccharide
LV: Lentivirus
MBP: Myelic basic protein
mi-MRI: Micro magnetic resonance imaging
NC: Normal control
PI3K: Phosphoinositide 3-kinase
NeuN: Neuronal nuclei
NF-200: Neurofilament-200
SCI: Spinal cord injury
SD: standard deviation
TNF-α: Tumor necrosis factor-α
WB: Western blot
