# Supplementary material for: Activation of glucagon-like peptide-1 receptor in microglia attenuates neuroinflammation-induced glial scarring via rescuing Arf and Rho GAP adapter protein 3 expressions after nerve injury
Source: Int J Biol Sci. 2022 Jan 16;18(4):1328–46. doi: 10.7150/ijbs.68974 (PMC8898359; doi:10.7150/ijbs.68974)
Supplement: Supplementary file 1 — Supplementary figures. [file ijbsv18p1328s1.pdf]

Figure S1.

(A) Fluorescent identification of microglia (IBA-1, green) and astrocyte (GFAP, red); Scale bar = 50 μm. **(B-C)** Flow cytometry identification of microglia (F4_80) and astrocytes (GFAP).

Figure S2.

(A) Western blotting for iNOS and ARAP3 in activated microglia treated with Ex-4, at concentrations of 0.2, 0.5, 1, and 2 μg/mL. **(B)** Quantitative analysis of iNOS and ARAP3 expressions.

Figure S3. Fluorescence images of GFP-LV-shRNA after transfection for 72 h, with titers of 10^5, 10^6, 5×10^6, and 10^7 TU/mL. HitransG A and HitransG P were used for 12 h as media, respectively.

Figure S4.

(A) Photographs of the spinal cord injury procedure. **(B)** Photographs of injured cords at 3 dpi, 7 dpi, and 28 dpi after 4% paraformaldehyde perfusion.

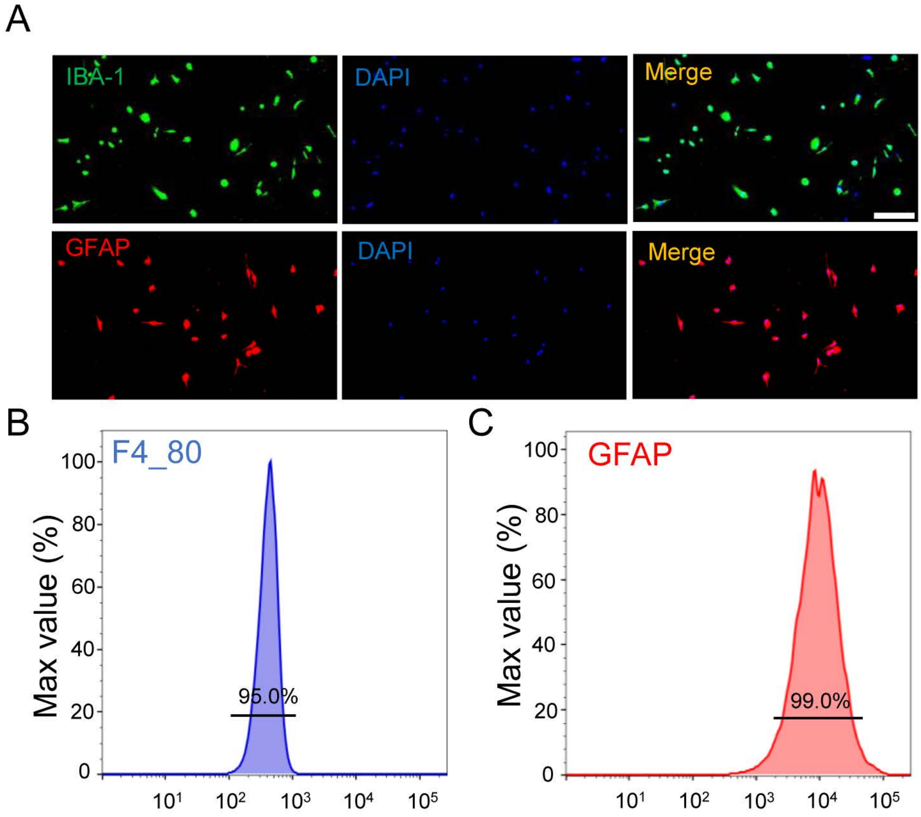
A
IBA-1
DAPI
Merge
GFAP
DAPI
Merge
B
F4_80
Max value (%)
95.0%
C
GFAP
Max value (%)
99.0%

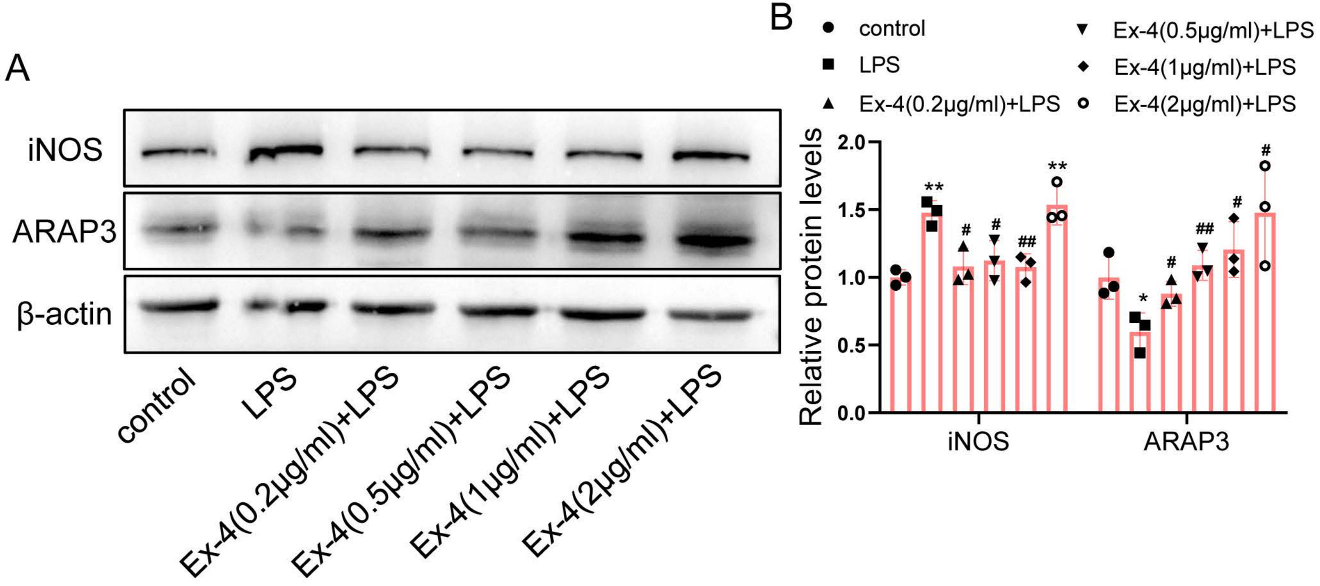
A
iNOS
ARAP3
β-actin
control
LPS
Ex-4(0.2μg/ml)+LPS
Ex-4(0.5μg/ml)+LPS
Ex-4(1μg/ml)+LPS
Ex-4(2μg/ml)+LPS
B
control
LPS
Ex-4(0.2μg/ml)+LPS
Ex-4(0.5μg/ml)+LPS
Ex-4(1μg/ml)+LPS
Ex-4(2μg/ml)+LPS
Relative protein levels
2.0
1.5
1.0
0.5
0.0
**
#
#
##
**
*
#
##
#
#
iNOS
ARAP3

A

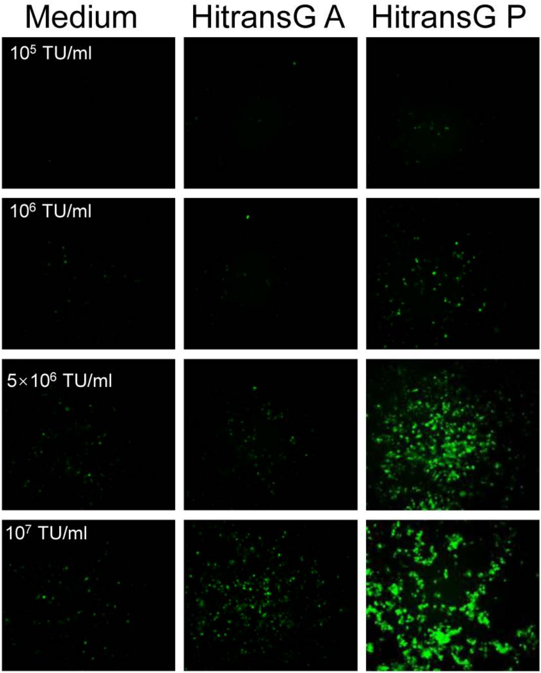

Medium
HitransG A
HitransG P
10^5 TU/ml
10^6 TU/ml
5×10^6 TU/ml
10^7 TU/ml

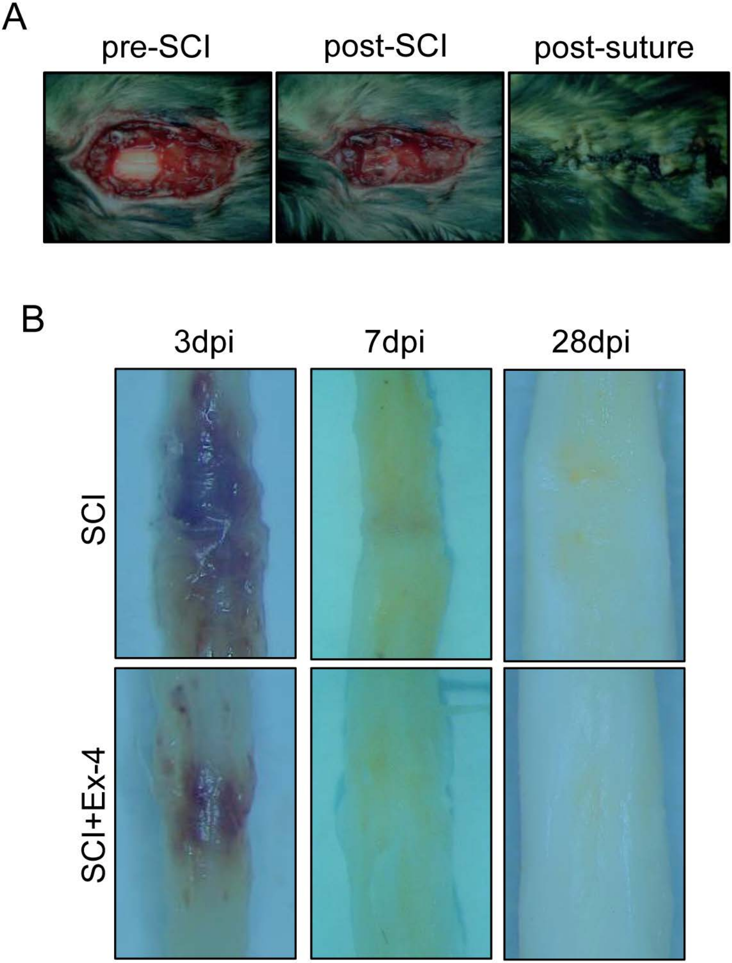

A
pre-SCI
post-SCI
post-suture
B
3dpi
7dpi
28dpi
SCI
SCI+Ex-4
